# Noradrenergic and cholinergic modulation of olfactory bulb sensory processing

**DOI:** 10.3389/fnbeh.2012.00052

**Published:** 2012-08-13

**Authors:** Sasha Devore, Christiane Linster

**Affiliations:** Computational Physiology Lab, Department of Neurobiology and Behavior, Cornell UniversityIthaca, NY, USA

**Keywords:** acetylcholine, noradrenaline, olfactory bulb, neuromodulation

## Abstract

Neuromodulation in sensory perception serves important functions such as regulation of signal to noise ratio, attention, and modulation of learning and memory. Neuromodulators in specific sensory areas often have highly similar cellular, but distinct behavioral effects. To address this issue, we here review the function and role of two neuromodulators, acetylcholine (Ach) and noradrenaline (NE) for olfactory sensory processing in the adult main olfactory bulb. We first describe specific bulbar sensory computations, review cellular effects of each modulator and then address their specific roles in bulbar sensory processing. We finally put these data in a behavioral and computational perspective.

## Introduction

Neuromodulators such as noradrenaline (NE), acetylcholine (ACh) and serotonin (5HT) serve important functions in sensory perception. Sensory perception, as much as other brain functions, needs to be regulated according to task demands, features of the sensory environment such as e.g., signal-to-noise ratio, and the animal's physiological state. Classically, each of these neuromodulatory systems has been linked to specific functions such as improvement of neuronal signal to noise ratios, attentional processes, general arousal, learning and memory (Sarter and Bruno, 1997; Schultz et al., 1997; Usher et al., 1999; Aston-Jones and Cohen, 2005; Sarter et al., 2005; Cools et al., 2008). However, a clear functional dissociation between the roles of these systems in sensory processing is not trivial and has not yet been elucidated. Each neuromodulator acts upon neurons in a variety of brain regions through a host of specific receptors resulting in changes of neural dynamics at both the cellular and network level [reviewed in Shea et al. (2008)]. These effects have been linked to alterations in sensory response magnitudes via altered signal to noise ratios, or changes in the temporal precision between afferent input and postsynaptic responses. At the network level, neuromodulation of cellular properties leads to a reduction of sensory thresholds, refinement of receptive fields to sharpen contrast, changes in oscillatory dynamics and synchronization, and increased plasticity.

We here review the role and function of two major neuromodulators, ACh and NE, for modulation of early olfactory perception; we focus our discussion on the olfactory bulb. The olfactory bulb network receives monosynaptic inputs from sensory neurons and has been directly implicated in sensory processing underlying behavioral plasticity (Cleland and Linster, 2005). This makes it a tractable and attractive system for the study of neuromodulatory regulation of sensory processing, as there are direct connections between neural and behavioral effects (Mandairon and Linster, 2009). The olfactory bulb is more than a sensory feedforward filter but rather actively shapes, and is actively shaped by, olfactory perception, a notion introduced more than 20 years ago by Freeman and colleagues (1982). A crucial component of this function is the central projections to the olfactory bulb, including cortical, sub-cortical and neuromodulatory noradrenergic and cholinergic projections [reviewed in Shipley and Ennis (1996)].

We here review the function and role of two neuromodulators, ACh and NE, for olfactory bulb function. We structure this review by describing specific bulbar sensory computations and how each modulator affects these known and hypothesized functions.

## Olfactory bulb network and processing

**Figure 1:**
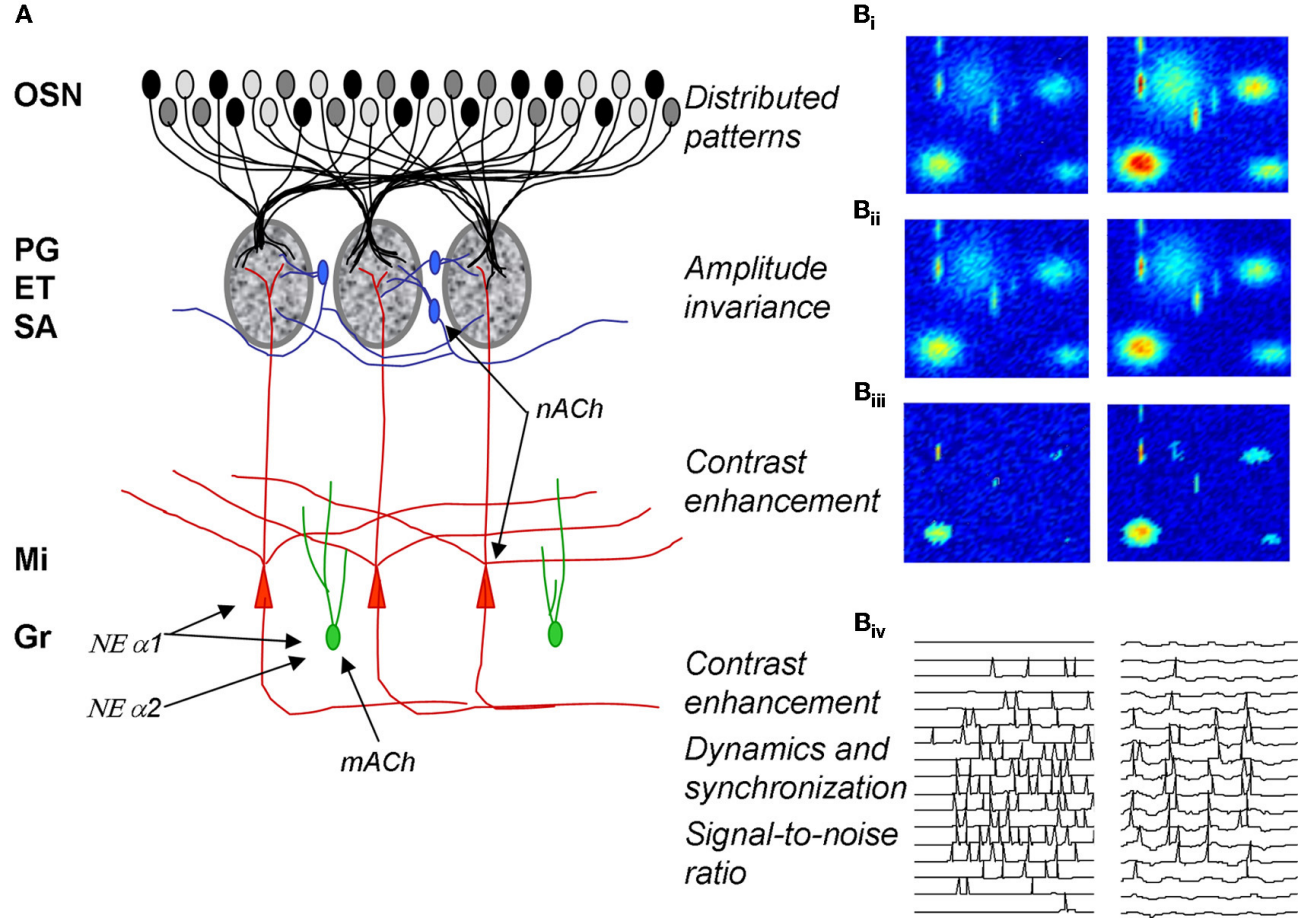
**Olfactory bulb processing. (A)** Schematic of olfactory bulb organization. Olfactory sensory neurons (OSN) expressing a common receptor and therefore exhibiting similar odor receptive fields project to common glomeruli in the olfactory bulb (MOB). Within this glomerulus, OSNs make excitatory synapses onto mitral and tufted cells, the primary output neurons of the MOB, as well as glomerular layer interneurons comprising periglomerular (PG) and external tufted (ET) cells. Most PG cells (~70%), and a third type of interneuron, short axon cells (SA), are not directly activated by OSNs. PG, ET, and SA cells form intricate networks within the glomerular layer that have been proposed to perform operations such as normalization, contrast enhancement and synchronization. In deeper processing layers mitral cells (Mi) interact with at least one other class of interneurons, granule cells (Gr). These provide extensive feedback and lateral interactions between mitral cells by interacting with their elongated secondary dendrites. This layer of processing is thought to be involved in creating olfactory bulb oscillatory rhythms and generating synchronized spike patterns. Noradrenergic inputs from the locus coeruleus activate three classes of noradrenergic receptors distributed across the MOB. NE α1 receptors are thought to be predominantly located on Mi and Gr cell bodies as well as secondary dendrites with a sparser distribution in the glomerular layers; NE α 2 receptors are mainly located on granule cells with a sparse distribution on Mi cell bodies and the glomerular layer; NE α receptors have been reported on Mi cell bodies and in the glomerular layer. Muscarinic ACh receptors are thought to be located on granule cells (mACh) whereas nicotinic ACh receptors are located on mitral and periglomerular cells (nACh). **(B)** Schematic depiction of glomerular layer functions and Mi cell activity patterns in response to odor stimulation. **(B_i_–B_iii_)** show simulated distributed odor responses at two concentrations with lower concentration in the left column and higher concentration in the right column. The two-dimensional simulations are color coded with red indicating high and dark blue low levels of activity. **(B_ii_)** shows the same patterns after amplitude-invariance processing has been performed by the network of local interneurons and **(B_iii_)** shows the same pattern after contrast enhancement. The patterns in **(B_iii_)** represent the end result of the glomerular computations transmitted to deeper layers by Mi cells. The details of these computations are given in Cleland and Sethupathy (2006); Cleland et al. (2007). **(B_iv_)** shows how spikes generated in Mi cells in response to activation patterns (left side) are transformed into sparser, oscillatory and highly synchronized spike patterns by the interactions with deeper interneuron networks (Mandairon et al., 2006; Escanilla et al., 2010).

The main olfactory bulb in rodents has been extensively described in a number of review articles. We briefly review the main neuronal types, their interactions as well as its hypothesized function in odor processing before describing the details of cholinergic and noradrenergic action onto these neurons (Figure 1).

Distributed patterns of activity in response to odorants are projected to the olfactory bulb glomeruli via olfactory sensory neurons (Figures 1A: OSN; 1B_i_). The olfactory bulb is believed to transform these incoming sensory data, performing a number of operations including normalization, contrast enhancement, and regulation of signal-to-noise before conveying the information to secondary olfactory structures (Cleland and Linster, 2005). The details of the neural networks involved in these computations have been described elsewhere: contrast enhancement (Cleland and Sethupathy, 2006) and concentration invariance (Cleland et al., 2007) involve networks of glomerular layer interneurons including periglomerular, external tufted and short axon cells.

At the level of glomerular processing, *changes in activation patterns*, i.e., which mitral cells are responsive to any given odorant are thought to be dominant and are largely shaped by the operations described above. Neuromodulatory inputs, mainly cholinergic and serotonergic and to a lesser degree noradrenergic, modulate and change these functions of the olfactory bulb, presumably adapting the computations to specific behavioral demands on the animal and to the nature of the chemical signal to be processed [reviewed in (Mandairon and Linster, 2009)].

In deeper layers of the bulb, modulation of *spike timing and synchronization* may affect contrast of odor representations (Spors and Grinvald, 2002; Urban, 2002; Migliore and Shepherd, 2008), signal-to-noise ratio (Linster et al., 2010), as well postsynaptic processing in secondary cortex (Linster and Cleland, 2012). Modulatory inputs on mitral and granule cells can potentially affect the dynamics of neural activity as well as spike timing and degrees of synchronization.

In summary, modulatory inputs have the capacity to actively regulate bulbar processing and have been shown to do so in a variety of paradigms and levels of investigation: glomerular modulation regulates contrast and normalization processing whereas in deeper layer modulatory inputs regulate contrast, oscillatory dynamics and synchronization properties. In the next section, we will review data pertaining to the cholinergic and noradrenergic modulation of these different networks in the main olfactory bulb of adult rodents.

## Cholinergic and noradrenergic modulation of bulbar processing

The OB receives extensive cholinergic inputs from the basal forebrain via the nucleus of the horizontal limb of the diagonal band of Broca (HDB) that innervates primarily glomerular and granule cell layers of the bulb (Heimer et al., 1990). ACh in the OB acts on both nicotinic and muscarinic receptors (Castillo et al., 1999; Ghatpande et al., 2006; Pressler et al., 2007); these are well segregated with a low degree of overlap, whereby nicotinic receptors are typically located within the glomerular and mitral cell layer while muscarinic receptors are located in granule cell layers. *In vitro*, activation of nicotinic receptors has been shown to depolarize mitral cells (Castillo et al., 1999); earlier *in vivo* studies also suggested that activation of nicotinic receptors would increase firing in periglomerular cells (Ravel et al., 1990). Muscarinic receptor activation increases the responsiveness of granule cells by direct depolarization (Castillo et al., 1999) as well as by transforming afterhyperpolarization into afterdepolarization (Pressler et al., 2007). Granule cells are therefore more excitable and the afterdepolarization uncovered by cholinergic inputs allows a sustained response once activated, thus changing the balance between excitation and inhibition in the deeper bulbar layers (Pressler et al., 2007). Overall, cholinergic inputs enhance both periglomerular inhibition of mitral cell primary dendrites as well as granule cell inhibition of mitral cell secondary dendrites. Simultaneously, mitral cells are depolarized, which counterintuitively renders them less sensitive to weak olfactory inputs while maintaining responsiveness to stronger inputs (Elaagouby and Gervais, 1992; Mandairon et al., 2006; Tsuno et al., 2008; D'Souza and Vijayaraghavan, 2012). The resulting effect of cholinergic modulation seems to be to enhance the specificity and temporal precision of mitral cell odor responses. Blockade of cholinergic muscarinic receptors decreased odor evoked beta oscillations *in vivo*, supporting this hypothesis (Chabaud et al., 2000). Accordingly, an *in vivo* study showed that odor responses of mitral cells are sparser when cholinergic modulation in the bulb is enhanced and that the population responses to chemically and perceptually overlapping odorants exhibit reduced overlap (Chaudhury et al., 2009).

The OB receives a dense noradrenergic projection from LC that terminates in all but the most superficial layers. NE fibers preferentially target the granule cell layer, and to a lesser extent the mitral cell layer (McLean et al., 1989). Each of the three major NE receptor subtypes (α1, α2, β) is expressed in multiple layers of the OB, and individual OB neurons appear to express multiple NE receptor subtypes. For example, mitral cells express all three NE receptor subtypes and granule cells express α1 and α2 receptors. *In vitro* data from adult rodent OB shows that activation of α1 and α2 receptors have opposing effects on granule cells: α2 activation decreases spontaneous IPSCs while α1 activation increases spontaneous IPSCs. Due to the fact that α2 receptors have a higher affinity for NE than α1 receptors, α2 effects dominate at very low concentrations of NE (Nai et al., 2009, 2010). At increasing concentrations of NE, α1 activation also depolarizes mitral cells (Nai et al., 2009). Presently, no conclusive data about β receptor activation in adult rodents exists. Overall, NE first decreases mitral cell inhibition and then increases inhibition onto mitral cells, while simultaneously rendering them more excitable. The net result appears to be an enhancement of network excitability, with NE resulting in enhanced oscillatory dynamics and synchronization between mitral cell spikes (Escanilla et al., 2010). Slice recordings in younger rodents show long-lasting, enhanced oscillations in the gamma frequency range when NE is added to the slice (Gire and Schoppa, 2008). *In vivo* recordings show that stimulation of the LC makes mitral cells more responsive to subthreshold olfactory nerve shocks (Jiang et al., 1996), indicating that mitral cell sensitivity to olfactory inputs is increased. This may be due to an effect of NE on mitral-granule cell interactions, as suggested by *in vivo* studies using glutamatergic activation of LC neurons (Okutani et al., 1998). More recently, it was shown that prolonged LC stimulation paired with prolonged odor stimulation depresses mitral cell odor responses and also triggers long term behavioral effects: after 24 h, the investigation response of rodents toward the paired odor is reduced (Zaborszky et al., 1986). In mice conditioned to an odorant, increases in levels of NE in the main olfactory bulb during presentation of the conditioned, but not a novel odor indicate a continuing role of NE in the expression of the association between the conditioned odor and the associated reward (Brennan et al., 1998). In sheep, NE inputs to the bulb play an important role in learning and recalling the odor of an ewe's lamb (Levy et al., 1990).

In summary, NE and ACh exert some common—depolarization of mitral and granule cells—and some differential—depolarization of PG cells—effects on bulbar networks. At the glomerular level, excitation of PG cells affects glomerular computations such as decorrelation of odor representations and concentration invariant representations—effects suggested to be modulated by cholinergic activation of nicotinic receptors (Linster and Cleland, 2002; Mandairon et al., 2006). At the level of the mitral-granule cell loop, both NE and ACh depolarize both cell types, and as a consequence would enhance bulbar dynamics and synchronization, presumably affecting signal to noise ratio (Linster et al., 2010) as well as postsynaptic processing of bulbar information (Linster and Cleland, 2012). However, the precise effects of ACh and NE on mitral and granule cells are different enough to create divergent resulting network effects, therefore suggesting differential functional roles for each. Accordingly, behavioral experiments show some differential effects of both bulbar neuromodulators as well as common effects; these are reviewed in the next section. Moreover, within individual systems, differential affinities for each receptor subtype can create non-linear dose-response curves (Nai et al., 2009, 2010) and perceptual consequences that may depend on the degree of activation of the neuromodulatory projection neurons themselves. Ultimately, computational modeling will be necessary to help differentiate between the possible perceptual consequences of modulating different aspects of the bulbar networks.

## Cholinergic modulation of contrast enhancement and perceptual discrimination in the glomerular layer

**Figure 2:**
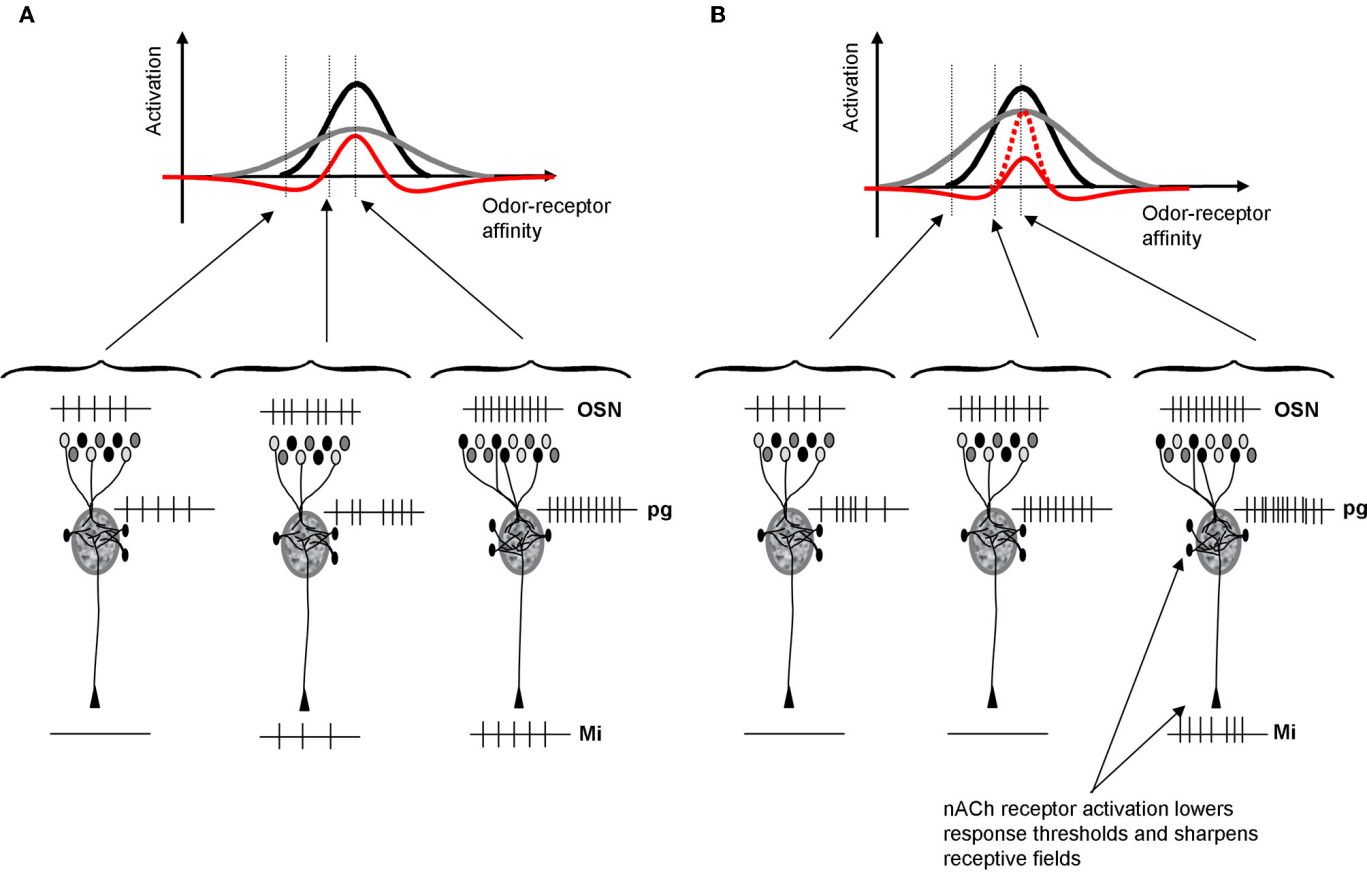
**Schematic depiction of cholinergic modulation of glomerular contrast enhancement. (A)** Within OB glomeruli, mitral and PG cells receive direct inputs from OSNs. PG cells have a higher input resistance and hence respond more quickly and saturate earlier than mitral cells. As a consequence, PG cells inhibit mitral cells in response to low affinity odorants but mitral cells override this inhibition in response to high affinity odorants. This microcircuit leads to contrast enhancement within each glomerulus, independent of location and receptive field (Cleland and Sethupathy, 2006; Cleland and Linster, 2012). The graph shows mitral (black) and PG (gray) cell activation levels as a function of varying the odor affinity between the receptor expressed in OSNs projecting to that glomerulus. PG cells inhibit mitral cells, resulting in a final activation (red) that is sharpened with respect to the incoming OSN signal. The schematic network below depicts the types of spike trains that might result from these interactions for odors located outside, at the lower end and in the middle of the OSNs receptive field. **(B)** Because ACh activates PG cells via nicotinic receptors, PG cell receptive fields (gray) are enlarged and as a consequence mitral cell receptive fields are sharpened (red). Nicotinic depolarization of mitral cells increases the amplitude of response within the positive part of the receptive field (dotted red line). As a consequence, mitral cell receptive fields are sharpened by ACh and contrast between odorants activating overlapping OSN populations is increased.

Distributed patterns of activity in response to chemical stimuli are transmitted to the olfactory bulb via OSN axons that terminate in the glomeruli of its input layer. It is clear from recent investigations that the perceptual qualities of odorants can be predicted, to a limited degree, from the patterns of activation that they evoke at the olfactory bulb input layer (Linster and Hasselmo, 1999; Linster et al., 2001, 2002; Cleland et al., 2002). Behavioral experiments manipulating the olfactory bulb have shown clear evidence that the relationship between primary olfactory representations and perception can be altered not only through experience but also through experimental manipulations of olfactory bulb function (Mandairon and Linster, 2009). Several pieces of evidence point to glomerular circuits as being at least partially responsible for contrast enhancement underlying perceptual discrimination: (1) modeling studies show that glomerular microcircuits are capable of performing contrast enhancement (Linster and Hasselmo, 1997; Cleland and Sethupathy, 2006; Linster and Cleland, 2009); (2) manipulations of nicotinic cholinergic receptors—located predominantly in the glomerular layer—enhance pairwise odor discrimination in rats (Figure 6A) and enhance mitral cell odor selectivity in *in vivo* recordings (Mandairon et al., 2006; Chaudhury et al., 2009); (3) glomerular activation patterns, mapped through 2DG and c-Fos or imaged through calcium visualization, largely predict the perceptual similarly of different odors in a number of behavioral tasks (Linster et al., 2001; Cleland et al., 2002). From these combinations of studies we can conclude that activation of nicotinic receptors in the glomerular and mitral cell layer modulates odor receptive fields of mitral cells in such a manner as to enhance contrast between representations (i.e., to decorrelate odor representations) (Figure 2).

Contrast enhancement in the glomerular layer is achieved by dendrodentritic interactions between PG and mitral cells (Figure 2). Both cell types receive the same inputs from OSNs; the amplitude of this input is determined by the affinity between the odor applied and the olfactory receptor expressed in cells projecting to that glomerulus. PG cells have a higher input resistance and hence respond faster and saturate earlier than mitral cells. As a consequence, mitral cells are inhibited in response to odor stimuli with weak affinity to the projecting receptor type: their odor receptive field is narrowed by PG cell input. When nicotinic receptors on PG and mitral cells are activated, two separate phenomena happen: (1) PG cells are more exited and inhibit mitral cells for a larger range of affinities and (2) mitral cells are depolarized and respond more strongly to those odorants that they are still responsive to. As a consequence, the output of the glomerular layer is more specific to particular subsets of odorants and the representations of odorants with overlapping affinity-patterns are rendered more distinct (Figure 2).

## Cholinergic modulation of synchronization and dynamics in the mitral-granule cell layers

**Figure 3:**
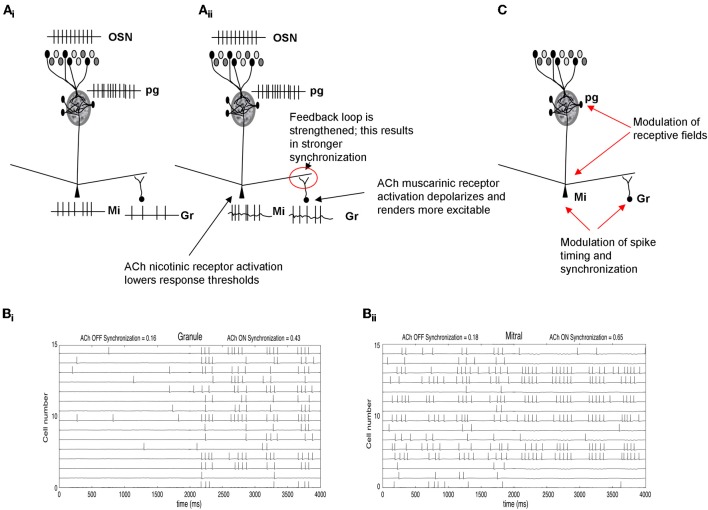
**Schematic depiction of cholinergic modulation of deeper layer synchronization and dynamics. (A_i_)** OSN activation by odorants is processed in the glomerular layer (Figure 2) and creates an odor-specific response in mitral cells. In the example shown here, mitral cells respond with a spike train and in turn activate granule cells though excitatory synapses. **(A_ii_)** Nicotinic ACh modulation depolarizes mitral cells and, at the same time, muscarinic receptor activation renders granule cells more responsive to input (Castillo et al., 1999; Pressler et al., 2007). Together, these enhance the excitatory-inhibitory feedback loop between these two groups of cells, increasing oscillatory dynamics and mitral cell spike synchronization. **(B)** Simulation of cholinergic effects in a computational model of olfactory bulb. **(B_i_)** The graph shows the membrane potential and action potentials of a subset of granule cells in the model. When ACh is “ON,” granule cells are depolarized and fire more easily in response to odor stimulation. **(B_ii_)** shows the corresponding membrane potential and action potentials of modeled mitral cells. When ACh is “ON,” mitral cells are more excitable due to activation of nicotinic receptors. The additional excitation is balanced by the higher inhibitory inputs from granule cells shown in **(B_i_)**. This balance does not significantly change mitral cell synchronization but because the loop is more strongly activated, both mitral and granule cells become more oscillatory and their action potentials are more synchronized (synchronization index calculates the number of synchronized spikes divided by the total number of spikes). The graph also shows the receptive field sharpening in mitral cells which is due to the nicotinic activation of PG cells described in Figure 2. **(C)** Summary of cholinergic modulation. ACh in the glomerular layer sharpens mitral cell receptive fields (Figure 2), whereas ACh modulation in deeper layers increases synchronization among odor responsive mitral cells.

Odor receptive fields, shaped by the glomerular computations described above, are then processed in the deeper bulbar layers, in which mitral cell spiking activity is further modulated by local interneurons before being conveyed to higher-order processing networks. In these deeper layers it is assumed that mitral cell odor receptive fields, i.e., whether and to what extent a mitral cell responds to an odorant, are already determined and that mainly spike timing is modulated (Cleland and Linster, 2005). Interactions between mitral and granule cells in the deeper bulbar layers are thought to produce bulbar oscillatory dynamics and synchronization (Bathellier et al., 2006; Brea et al., 2009). Synchronization is important for olfactory processing and learning (Stopfer et al., 1997) and has been associated with olfactory discrimination capabilities in genetically modified mice (Nusser et al., 2001). A study by Beshel et al. (2007) showed that bulbar high frequency oscillations are enhanced when tasks demands increase showing that not only oscillatory synchrony is important for odor perception but that it can be regulated by the task difficulty (Beshel et al., 2007). Previous studies by Ravel and colleagues showed that slower oscillations in the beta range are modulated during olfactory learning and that their existence in the olfactory bulb can be predictive of how well a discrimination task has been learned (Martin et al., 2004, 2006). These slower waves are modulated by cholinergic inputs under certain behavioral states (Elaagouby et al., 1991; Tsuno et al., 2008). Computational modeling studies demonstrate that synchronization patterns among mitral cells can also affect the read out of bulbar activity by the next layer of neurons in higher order olfactory processing networks (Linster and Cleland, 2001, 2012). In fact, there appears to be a lower bound on the number of synchronized spikes required to drive piriform cortex pyramidal cells (Davison and Ehlers, 2011; Franks and Isaacson, 2006). Thus, changes in synchronization patterns at the output of the OB affect cortical readout and therefore odor perception. Cholinergic inputs to mitral cells, acting on nicotinic receptors, and on granule cells, acting on muscarinic receptors, activate the oscillatory feedback loop between these two cell types and as a consequence can increase oscillatory dynamics and synchronization (Figure 3). Hypothetically this can lead to changes in the learning rate of olfactory information (Linster and Cleland, 2012; deAlmeida et al., submitted).

Interestingly, in our previous behavioral experiments, modulation of muscarinic receptors was not effective by itself but did enhance the effect of nicotinic receptor modulation on olfactory discrimination (Mandairon et al., 2006). On the other hand, a more difficult delayed match-to-sample task, in which an odor has to be memorized and compared to a second, was strongly affected by blockade of only muscarinic receptors (Ravel et al., 1994). Apparently, synchronization patterns become more important for perception as task demands become more difficult either by having highly perceptually similar odors (Stopfer et al., 1997) or because more complicated processes than odor discrimination are involved, especially memorization and comparison of stimuli across time. Indeed, tasks in which a small temporal delay between stimulus and decision is involved may rely on bulbar plasticity which could be facilitated by muscarinic receptor modulation as shown in other systems (Weinberger, 2003; Roberts et al., 2005; Butt et al., 2009), however this hypothetical role of muscarinic receptors in the bulb remains to be tested.

In summary, in deeper layers of the bulb, cholinergic inputs activate the mitral-granule cell feedback loop, thereby possibly increasing synchrony among odor-responsive mitral cells (Figure 3; Pressler et al., 2007). Increased afterdepolarization in granule cells renders them more excitable and can lead to sustained firing, further increasing the oscillatory dynamics and synchronization properties. The increased synchrony would lead to more specificity in cortical responses, which in turn allows faster learning (Linster and Cleland, 2012). Thus, rather than changing broad odor receptive fields of mitral cells, as described for glomerular modulation, cholinergic modulation in the deeper layers of the bulb changes synchronization patterns and with it the learning of odor stimuli in higher-order processing networks.

## Noradrenergic modulation of odor sensitivity

**Figure 4:**
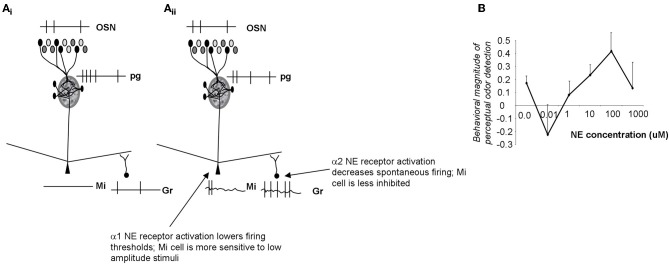
**Schematic depiction of noradrenergic modulation of odor sensitivity. (A_i_)** OSN inputs are processed in the glomerular layer and result in activation of a subpopulation of mitral cells. The spiking response of these active cells is further influenced by inhibitory inputs from granule cells. In the example shown here the applied stimulus was below threshold in amplitude, resulting in no spiking response in mitral cells. **(A_ii_)** NE α2 activation (at very low NE concentrations) decreases granule cell spontaneous activity, and disinhibits mitral cells (Nai et al., 2009, 2010). Mitral cells are more responsive to odorants and to low amplitude electrical stimulation (Jiang et al., 1996; Ciombor et al., 1999). At higher NE concentrations, α1 receptors are activated, granule cell spontaneous activity increases and mitral cells are inhibited (Nai et al., 2009, 2010); at the same time activation of mitral cell α1 receptors depolarizes mitral cells, rendering them more responsive to stimulation. As a consequence, α1 activation leads to increased sensitivity while preserving discrimination (Linster et al., 2010). **(B)** Behavioral experiments show that odor detection at near-threshold concentration initially decreases, then increases with increasing concentrations of NE, following the non-linearities observed in brain slice recordings (Escanilla et al., 2010; Linster et al., 2010). The graph shows the degree of odor detection measured in a spontaneous odor detection task as a function of NE concentration infused directly into the OB of adult rats.

Noradrenergic inputs act on α1 receptors on mitral cells as well as α1 and α2 receptors on granule cells. Brain slice and *in vivo* data shows that α1 receptor activation modulates the response of mitral cells to weak olfactory nerve stimulation, rendering them more sensitive to near-threshold stimuli (Jiang et al., 1996; Ciombor et al., 1999). On the other hand, activation of α1 receptors also increases the strength of inhibitory inputs to mitral cells from granule cells, thereby increasing inhibition on mitral cells. α2 receptors decrease inhibitory outputs from granule cells. Because α2 receptors are activated at lower NE concentrations, mitral cells are first disinhibited, then both inhibited and excited as NE concentration increases (Figure 4). This leads to mitral cells becoming more sensitive to inputs at low NE concentrations, staying more sensitive to input at medium NE concentrations while also responding more specifically because of increased granule cell inhibition, and becoming less sensitive again at higher NE concentrations when α2 effects are overshadowed by α1 effects (Figure 4; Escanilla et al., 2010; Nai et al., 2010). Behavioral experiments showed that when NE was infused into the OB, perceptual threshold for odorants could be decreased by several orders of magnitude in a dose-dependent manner (Figures 4, 6B; Escanilla et al., 2010). At the same time, discrimination thresholds were also decreased, showing that the increased sensitivity, which could be explained by increased activation of mitral cells alone, was accompanied by a second process preserving discrimination capabilities despite the overall increase in sensitivity (Escanilla et al., 2010). Accordingly, modeling studies in which these two effects can be separated show that if α1 only acts on mitral cells, sensitivity is increased at the expense of discrimination (Linster et al., 2010).

Overall, activation of NE receptors on mitral and granule cells changes the excitability of mitral cells in a non-linear dose dependent relationship that is reflected in the behavioral response (Figure 4; Linster et al., 2010). The receptive field of mitral cells is pre-determined by OSN inputs and glomerular processing; α1 activation on mitral cells renders them more responsive to weak inputs whereas the accompanying increase of granule cell firing preserves discrimination.

## Modulation of signal-to-noise ratio

**Figure 5:**
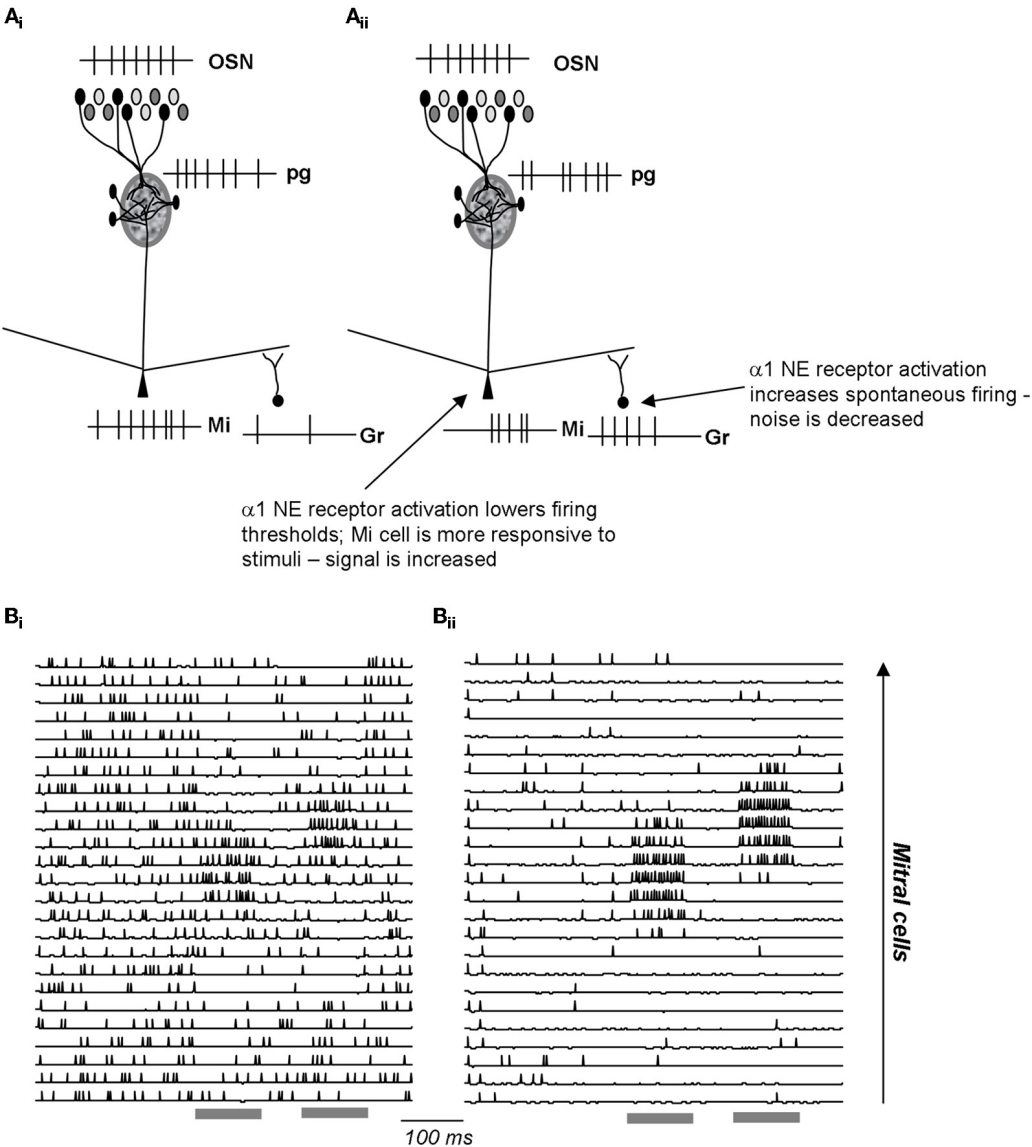
**Schematic depiction of noradrenergic modulation of signal-to-noise ratio in the EPL. (A_i_)** OSN inputs are processed in the glomerular layer and result in activation of a subpopulation of mitral cells. The spiking response of these active cells is further influenced by inhibitory inputs from granule cells. **(A_ii_)** NE α1 activation increases granule cell spontaneous activation, resulting in less spontaneous in activity in the connected mitral cells. At the same time, a1 activation lowers mitral cell response thresholds, rendering them more sensitive to odor inputs. As a consequence, α1 activation leads to increased signal-to-noise ratio. **(B_i_–B_ii_)** Simulations [from Linster et al. (2010)] of a1 effects in the OB network. The graphs show the membrane potential and action potentials of modeled mitral cells during spontaneous and odor triggered activity. Odor stimulation is shown in grey below the traces. In the unmodulated state **(B_i_)**, spontaneous activity is high and odor triggered activity comparatively low. In the modulated state **(B_ii_)**, spontaneous activity is low (low noise), and odor triggered response comparatively strong (high signal).

**Figure 6:**
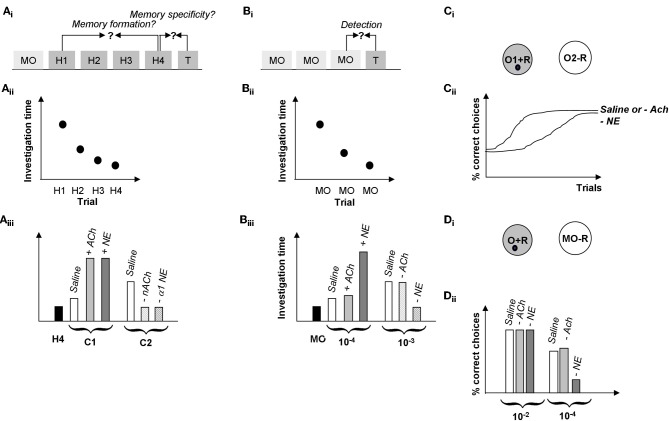
**Summary of behavioral results. (A)** Spontaneous odor discrimination task. **(A_i_)** Rats are first habituated to an odor during four habituation trials (H1-H4). A significant decrease in odor investigation time over the course of these trials indicates the formation of an odor memory. Rats are then presented with a test odor (T) of variable similarity to the habituated odor; a significant increase in investigation indicates that rats discriminate between the habituated and the test odor (Cleland et al., 2002). **(A_ii_)** Manipulations of cholinergic or noradrenergic modulation in the olfactory bulb in these experiments did not affect rats' formation of the odor memory; in our hands, all rats habituated to the odorants over the course of several experiments manipulating Ach or NE activity in the OB (Mandairon et al., 2006, 2008; Escanilla et al., 2010). **(A_iii_)** Manipulation of ACh and NE activity in the OB modulated odor discrimination in this task. In summary, test odorants NOT discriminated by control rats (C1, usually a straight chain aldehyde with a 1 carbon difference to the habituated odor), could be discriminated when increasing cholinergic (+ACh) or noradrenergic (+NE) modulation in the OB (Mandairon et al., 2006, 2008; Escanilla et al., 2010). On the other hand, test odorants discriminated by control rats (two or more carbons removed from the habituated odorant) are less well-discriminated when nicotinic ACh receptors (−nACh) or α1 NE receptors (−α1 NE) are blocked (Mandairon et al., 2006, 2008; Escanilla et al., 2010). **(B)** Spontaneous odor detection task. **(B_i_)** Rats are first habituated to the odor carrier, mineral oil during three or four habituation trials (MO). Rats are then presented with a test odor (T) of variable concentrations; a significant increase in investigation indicates that rats detect the odorant at that concentration. **(B_ii_)** Manipulations of cholinergic or noradrenergic modulation in the olfactory bulb in these experiments did not affect rats' response to mineral oil. **(B_iii_)** Manipulation of NE but not ACh activity in the OB modulated odor discrimination in this task. In summary, test odorant concentrations NOT detected by control rats (10^−4^ Pa) were detected by rats infused with additional NE into their bulbs (+NE), in contrast, infusion of the non-specific cholinergic agonist CCh (+ACh) had no effect on odor detection. Similarly, odor concentrations easily detected by control rats (10-2 Pa) were detected by rats with blocked ACh receptors (−ACh) but not rats with blocked NE receptors (−NE). The blockade of NE receptors was shown to be specific to α1 receptors (Mandairon et al., 2006, 2008; Escanilla et al., 2010, 2011). **(C)** Rewarded discrimination task. **(C_i_)** In this task, rats are presented with two odorized cups and learn to dig for a reward in the rewarded odor (O1 + R) and to ignore the non-rewarded odor (O2–R) over the course of 20 trials (Cleland et al., 2002). **(C_ii_)** Rats with cholinergic receptors blocked (−ACh) learn a discrimination between highly similar odorants at similar rates to saline infused control rats whereas rats with all NE receptors blocked (−NE) made significantly more mistakes over the course of a session (Mandairon et al., 2006, 2008; Escanilla et al., 2010, 2011). The graph shows the percentage of correct choices made by the rats over the course of 20 trials as a function of drug treatment and test odor concentration. **(D)** Rewarded detection task. **(D_i_)** In this task rats are trained to retrieve a reward from an odorized cup (O + R) presented at the same time as an unodorized cup (MO–R). Detection thresholds are measured by using variable near-threshold concentrations for the odorized cup and recording the percentage of correct choices made. **(D_ii_)** In this task, rats with NE receptors blocked (−NE) were significantly impaired at detecting low concentration odorants as compared to saline infused control rats and rats with cholinergic receptors blocked (−ACh). The graph shows the average number of correct trials during a session as a function of drug treatments (Escanilla et al., 2011).

Signal-to-noise ratio in sensory systems can be defined as the ratio between the number of spikes evoked by a sensory input and the total number of spikes during a comparable time frame (Linster and Hasselmo, 1997). NE specifically has been associated with modulating the signal-to-noise ratio in sensory systems (Usher et al., 1999; Aston-Jones and Cohen, 2005), however, attentional processes associated with ACh could also be interpreted as changes in signal-to-noise ratio in certain cases (Sarter and Bruno, 1997; Sarter et al., 2005). Because NE infusions decreased odor detection thresholds in rats considerably (Escanilla et al., 2010), we tested computationally if this could be attributed to changes in signal-to-noise ratio in the olfactory bulb. α1 activation of mitral cells enhances their response to odor stimulation, increasing “signal”, whereas increased spontaneous release of GABA from granule cells, as described experimentally, limits spontaneous activity, thereby decreasing “noise” (Figure 5). Overall this combination of effects results in an increase of signal-to-noise ratio in a dose-dependent manner (Escanilla et al., 2010). As a consequence, the pattern of activity during low concentration stimulation is statistically more different from spontaneous activity, resulting in better detection of low amplitude stimuli. Because ACh also activates both granule and mitral cells, it could have similar effects on signal-to-noise ratio; however, in our hands, increasing cholinergic modulation in the OB did not affect perceptual detection of low concentration stimuli [see summary of behavioral effects in Figure 6, Escanilla et al. (2011)]. While this discrepancy could be accounted for if rats differentially engage their cholinergic and noradrenergic systems during detection, a more attractive hypothesis is that although the cellular effects of NE and ACh on granule cells seem similar, they are not identical and may, in fact, subserve distinct functional roles.

## Functional differences between cholinergic and noradrenergic modulation related to differences in cellular effects

**Figure 7:**
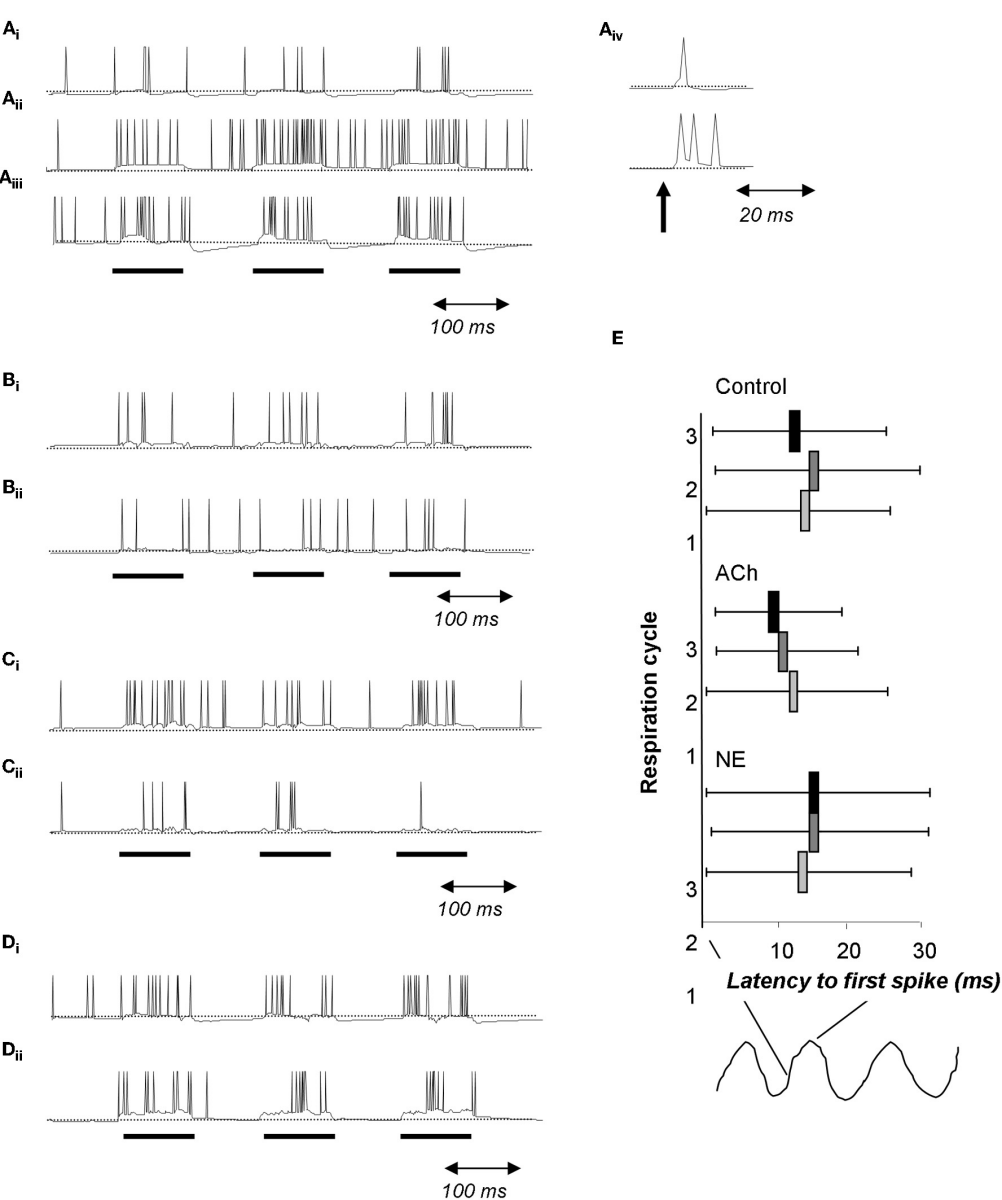
**Cholinergic and noradrenergic modulation of granule cells. (A)** Simplified integrate and fire model of a granule cell. **(A_i_)** Baseline condition. The cell is driven with 100 ms pulses of current injection separated by 100 ms. **(A_ii_)** Activation of muscarinic receptors changes afterhyperpolarization into afterdepolarization; as a consequence the cell is more active during the interstimulus intervals. **(A_iii_)** Activation of α1 NE receptors depolarizes the cell leading to an increase in the evoked response followed by a strong hyperpolarization during the interstimulus intervals. **(A_iv_)** When driven by a short current pulse (1 ms), the baseline granule cells emits a single spike followed by a small after hyperpolzarization (upper trace) whereas the granule cell under muscarinic activation responds with a spike followed by a afterdepolarization capable of triggering additional spikes. **(B)** In this simulation, a single mitral cell **(B_ii_)** receives 100 ms current injections, drives an attached granule cell and is inhibited by this same granule cell **(B_i_)**. **(C)** In this simulation the granule cell undergoes modulation of its after hyperpolarization in response to muscarinic receptor activation similar to that shown in A. Note the increased firing of the granule cell **(C_i_)** accompanied by a decrease in firing of the connected mitral cell **(C_ii_)**. **(D)** Effect of a1 NE activation on a single mitral **(D_ii_)** and granule **(D_i_)** cell loop. Both cells are more excitable and fire more during the current injections is observed. **(E)** Effect of cholinergic and noradrenergic modulation of granule cells on mitral cell spike latency. The graph shows the average latency to first spike during respiration cycles 1, 2, and 3 as well as the standard deviation of the latency. Under control conditions, the latency does not vary across respiration cycles (Control). When muscarinic receptors on granule cells are activated, the average latency as well as the standard deviation is decreased over successive respiration cycles (ACh). In contrast, activation or noradrenergic a1 receptors on granule cells does not affect spike latency nor its standard deviation (NE).

While the cellular effects of ACh and NE on bulbar cells can look similar on the surface, they differ in specific details of channels modulated and precise effects on neural behavior. In the glomelular layer, ACh acting on nicotinic receptors is strongly implicated in regulating periglomerular inhibitory microcircuits performing contrast enhancement and receptive field decorrelation (Linster and Cleland, 2002; Mandairon et al., 2006). At present, there is insufficient data on the glomerular-layer effects of NE acting on β receptors, although this is an active area of research. Deeper in the bulb, mitral cell spontaneous firing frequency is enhanced through inward currents induced by nicotinic receptor activation (Castillo et al., 1999). Similarly, mitral cell activation of NE α1 receptors induces an inward current in mitral cells that is accompanied by an increase in firing probability in response to near threshold stimulation (Nai et al., 2009). In granule cells, NE activation of α2 receptors reduces, and activation of α1 receptors increase spontaneous IPSCs onto mitral cells (Nai et al., 2009, 2010). As a consequence, mitral cells are disinhibited and therefore more responsive to stimulation at lower NE concentrations, and are depolarized but receive more inhibition at higher NE concentration, resulting in mitral cells that are more responsive to weak stimuli yet exhibit enhanced specificity (Figure 4). On the other hand, ACh activation of muscarinic receptors on granule cells reduces spontaneous IPSCs onto mitral cells (Castillo et al., 1999), but prolongs granule cell responses to direct activation by changing afterhyperpolerization into afterdepolarization (Pressler et al., 2007). This leads to granule cells that can be continuously active over the course of a respiratory cycle in response to odor stimulation (Figure 7A), in contrast to noradrenergic modulation which makes cells more active overall but does not affect afterpotentials (Figure 7). As a consequence, granule cells are less entrained to the respiratory rhythm under cholinergic modulation than under noradrenergic modulation (Figure 7A). Ultimately, cholinergic modulation of granule cells changes spike timing of mitral cells, leading to a significant decrease in the latency and variability of the first spike over successive respiration cycles. Under control conditions, the latency to first spike does not change over the course of the first three respiration cycles (Figures 7B,C,D). When granule cell muscarinic receptors are activated, granule cells fire in a more sustained manner over the course of the three first respiration cycles and mitral cell latency to first spike is significantly reduced during the second and third respiration cycle (Figure 7E). Noradrenergic modulation of granule cells does not have an effect on spike latencies (Figure 7E). The decrease of spike latency, accompanied by less variability in spike latency in response to muscarinic receptor activation could potentially contribute to a more precise latency code, driving higher-order processing networks in a more odor-specific manner (Linster and Cleland, 2012). Behaviorally, modulation of NE receptors seems to be implicated in low odor concentration detection and discrimination (Figures 6A,B,C) whereas modulation of cholinergic receptors seems to be involved in discrimination between highly similar odorants (Figure 6A) but not as clearly in low odor concentration detection or discrimination (Figures 6B,C).

## Conclusions

Central and modulatory inputs to primary sensory structures allow for flexibility and regulation of sensory processing according to behavioral demands. The olfactory bulb, for example, receives more efferent than afferent inputs (Shipley and Ennis, 1996) and its processing is heavily influenced by these central inputs [reviewed in Mandairon and Linster (2009)]. Olfactory bulb activity can be directly correlated to odor perception (Mandairon and Linster, 2009); perceptual tasks can modulate bulbar processing and vice versa. Neuromodulatory inputs such as ACh and NE have specific cellular effects reflected in behavioral effects, which can be best characterized through a combination of cellular, *in vivo*, behavioral and modeling approaches.

We have reviewed the role and function of two major neuromodulators, ACh and NE, for modulation of main olfactory bulb processing in adult rodents and early olfactory perception. Each modulator affects bulbar processing through a variety of cellular mechanisms acting on multiple circuits within the OB. Presumably, each modulator has a specific functional role, although their experimentally observed effects overlap substantially, making a clear dissociation difficult. Although these two neuromodulatory systems may exhibit differences in activation dynamics during specific behaviors, both systems are typically at least somewhat engaged during the waking state. Ultimately, it will be important to elucidate interactions between these neuromodulatory systems, either by direct projections between them or local effects, as well as additive effects. Furthermore, neuromodulatory systems project broadly throughout the central nervous system and target numerous structures beyond early sensory areas. Another key question that remains to be addressed is to understand how neuromodulatory inputs regulate interactions between early and higher-order sensory and cognitive networks. Indeed, it has been shown computationally that manipulating even subtle features of OB output can lead to dramatic changes in the response of higher-order processing networks (Linster and Cleland, 2012), which in turn feed back to influence processing in the bulb (Shipley and Ennis, 1996). Neuromodulatory systems such as noradrenalin and ACh are ideally situated to regulate not only processing within individual networks, but the interactions between them, which ultimately underlie complex behaviors.

### Conflict of interest statement

The authors declare that the research was conducted in the absence of any commercial or financial relationships that could be construed as a potential conflict of interest.
